# Macrophage Activation Syndrome secondary to Systemic Juvenile Idiopathic Arthritis: A Case Report

**DOI:** 10.31729/jnma.7019

**Published:** 2021-11-30

**Authors:** Rishikesh Kafle, Anwesh Bhatta, Sumit Gami, Abhin Sapkota, Dipesh Sharma, Arabindra Yadav, Vijaya Kumar Chikanbanjar

**Affiliations:** 1 Department of Pediatrics, Kathmandu Medical College Teaching Hospital, Sinamangal, Kathmandu, Nepal; 2 Patan Academy of Health Sciences, Lalitpur, Nepal; 3 Vayodha Hospitals Private Limited, Balkhu, Kathmandu, Nepal; 4 Karuna Hospital, Budanilkantha, Kathmandu, Nepal

**Keywords:** *juvenile arthritis*, *macrophage activation syndrome*, *steroids*

## Abstract

Macrophage activation syndrome is a rare but a life threatening condition commonly associated with Systemic Juvenile Idiopathic Arthritis. Its clinical presentation includes fever, hepatosplenomegaly, hypertriglyceridemia, hypofibrinogenemia, hyperferritinemia and impaired liver enzymes. The symptoms are alarming yet non-specific and often lead to a delayed diagnosis. A 12 year male presented with a history of intermittent fever and was started on antibiotics but failed to respond after several days of hospital stay. After a series of investigations to rule out multiple diagnoses he was diagnosed as a case of Macrophage Activation Syndrome secondary to Systemic onset Juvenile Arthritis and was treated with steroids.

## INTRODUCTION

Macrophage Activation Syndrome (MAS) is a clinical condition characterised by uncontrolled activation and proliferation of the T lymphocyte and macrophages.^1^ It is a form of hemophagocytic lymphohistiocytosis seen in heterogenous group of diseases such as viral infections, neoplasm and rheumatic disorders.^2^ The specific aetiology of MAS in not well mentioned but it often presents with Systemic Juvenile Idiopathic Arthritis (SoJIA).^3^ Diagnosis is often delayed as there is presence of overlapping symptoms of other common clinical conditions. Prompt diagnosis changes the treatment course and prevents fatal outcome. Here, we present a case of 12 year male with MAS secondary to SoJIA.

## CASE REPORT

A 12 years male from Charikot, Dolakha presented to our hospital with history of intermittent fever for one month duration, initially lasting for 1-2 days with 1-2 spikes with maximum recorded temperature of 101°F, responding well to paracetamol, not associated with chills, rigor or sweating and with intervening afebrile intervals of 3-4 days. However, six days prior to presentation high grade fever was noticed daily with 2-3 spikes with maximum recorded temperature of 104°F associated with chills, rigors and sweating with no diurnal variation, relieved by paracetamol for 6-8 hrs. Apart from fever there was no other significant positive history.

Initially, he was admitted for 3 days at a hospital in Dolakha and was treated with cefixime at nonenteric dosage but later switched to intravenous (IV) ceftriaxone and tab. azithromycin as fever persisted. Investigations were sent with suspicion of enteric fever most of which were unremarkable except for Haemoglobin (Hb): 10.8g/dl, Total leukocyte count (TLC): 2600/mm3, Neutrophils (N) 61, Lymphocytes (L) 35, Platelets: 120000, and was referred to our centre for persistence of fever.

At presentation, the child was ill looking, with moderate pallor and mild hepatosplenomegaly. He was admitted in the isolation ward awaiting the Reverse-transcriptase polymerase chain reaction (RT-PCR) report for Coronavirus disease (COVID-19) and continued on azithromycin and ceftriaxone, with initial investigations unremarkable as prior apart from Hb: 9.8g/dl and TLC: 1700/mm3. RT-PCR was negative for COVID-19 and as there were no respiratory symptoms and chest x-ray was also normal, with persistent fever despite seven days of IV ceftriaxone, zoonotic panel was sent and he was started on doxycycline. Zoonotic panel was negative, but blood culture was positive for Coagulase Negative Staphylococcus, so IV meropenam and vancomycin were started following sensitivity panel along with doxycycline.

On 5^th^ day of admission, ecchymotic rashes was noted on bilateral mid thigh measuring around 10x8 cm which were non-tender with smooth and clear margins along bilateral inguinal lymphadenopathy measuring approx 2.5 cm. Fever was persistent even after 72 hours of initiation of IV meropenam and vancomycin and doxycycline was stopped at seven days. So, all the investigations were repeated, with Hb: 9.8g/dl, TLC: 2100/mm, Erythrocyte sedimentation rate (ESR): 12, Packed cell volume (PCV): 24.2, Lactate dehydrogenase enzyme (LDH): 1887 and rest being unremarkable. Serology was non-reactive, Prothrombin time(PT/INR), Activated partial-thromboplastin time (APTT) and Antinuclear antibodies (ANA) were negative. Peripheral blood smear was unremarkable except for microcytic hypochromic anemia and low Absolute neutrophil count (ANC).

Echocardiography and ultrasound sonography test (USG) of abdomen was planned to rule out infective endocarditis, and intra-abdominal abscess and Mantoux test was done to rule out tubercular infection. USG abdomen showed borderline splenomegaly (120.3mm) and mild ascites. Mantoux was negative and echocardiography revealed mild pericardial effusion with normal left ventricular systolic function.

At this point, as leucopenia was still persistent with other suggestive findings so treatment was continued in line of sepsis with IV meropenam and vancomycin. Infective endocarditis was ruled due to absence of murmur and vegetations along with Systemic lupus erythematosus (SLE) and other connective tissue due to negative ANA.

On 10^th^ day of admission, fever was persistent, with no improvement and no new symptoms and signs.

Oral chloroquine was added for three days and bone marrow biopsy was sent to rule out leukemias and lymphomas.

On 12^th^ day of admission fever was persistent, and he was on day 8 of IV meropenam and IV vancomycin. Drug fever was suspected with prolong use of antibiotic, so all antibiotics were stopped and fever charting was done for 48 hrs. Even after 48 hrs of stoppage of antibiotic, fever was persistent with daily 2-3 spikes so high dose aspirin (75mg/kg/day) was started in view of suspected Atypical Kawasaki disease and Juvenile Rheumatoid arthritis. The bone marrow aspiration report was positive for microcytic hypochromic anemia along with leucopenia with normal cellularity and no features of neoplasm but numerous well-differentiated macrophages actively phagocytosing hematopoietic cells were prevalent.

With above findings, malignancy was ruled out and with evidence of serositis (ascites and pericardial effusion), anemia and leukopenia and clinical evidence of fever with hepatosplenomegaly, MAS was suspected and serum ferritin, triglycerides, LDH and fibrinogen levels were ordered. All investigations including triglycerides: 378 mg/dl (<200), LDH: 2375 IU/L, Ferritin: >1650 ng/ml, Fibrinogen level: 125mg/dl, were suggestive so a diagnosis of MAS secondary to SoJIA was made and treatment with IV methylprednisolone. After 2 dosages of methylprednisolone patient improved with defervesce and he was discharged.

## DISCUSSION

MAS, also known as secondary hemophagocytic lymphohistiocytosis (HLH) is a systemic hyper-inflammatory state resulting from abnormal natural killer and T-cell activation leading to a cytokine storm and ultimately the over-activation of macrophages producing an excessive inflammatory response and hyper-secretion of cytokines such as interferon gamma, tumor necrosis factor, interleukin (IL)-1, IL-6, IL-10, IL-12, IL-18, and macrophage colony-stimulating factor. The estimated prevalence of MAS in SoJIA is approximately 10%.^3,4^

Various triggering events like bacterial and viral illness (Epstein - Barr virus, hepatitis A infection, Cytomegalovirus) and drugs like non-steroidal anti-inflammatory drugs, gold salts, Sulfasalazine, Methotrexate and even Etanercept have been described to be associated with MAS. For instance, a case of MAS was described secondary to Epstein-Barr virus (EBV) infection, in a patient with SoJIA, confirmed by typical clinical and laboratory manifestations, myelogram and positive serology against EBV.^5^ However, MAS can occur without any identifiable triggers as was probably seen in our case. EBV and tuberculosis, are some of the common infections associated with MAS, which were excluded in our patient by appropriate tests.

MAS can have a very aggressive clinical course with sudden onset non-remitting high fever, bilateral cervical lymphadenopathy, hepatosplenomegaly, and altered mental status as described in a case report from NewDelhi.^6^ Our case however had milder presentation with persistent fever, mild hepatosplenomegaly and bilateral inguinal lymphadenopathy along with development of ecchymotic rashes on the bilateral thighs without alteration iof mental status at any stage.

The clinical feature of MAS may mimic a flare of SoJIA. SoJIA can present with quotidian fever, arthritis, lymphadenopathy, hepatosplenomegaly, serositis and evanescent rash. In the above mentioned case by Vishwanath VK et al. MAS was missed initially because arthritis was present with elevated ESR and there were no mental change. Thus, the patient was inadequately treated leading to death of the patient. Death rates of 11-22 % are have been reported.^7^

The laboratory findings in our patient were of pancytopenia, elevation of serum liver enzymes, hypofibrinogenemia, hypertriglyceridemia and hyperferritinemia which brought up suspicion of MAS and the bone marrow examination showed numerous well-differentiated macrophages actively phagocytosing hematopoietic cells. The rarity of the condition, variable clinical presentation, significant overlap with other clinical conditions and the time needed for diagnostic testing are some of the many challenges that delayed the diagnosis of the our case. Only after 14^th^ day of admission were we able to rule out the possible infective and neoplastic causes for the persistent fever in the patient.

Our patient fulfilled six criterias including fever >38.5°C, splenomegaly, cytopenias (anemia and thrombocytopenia), hypertriglyceridemia (with hypofibrinogenemia), hyperferritinemia and haemophagocytosis out of the eight needed for diagnosis of HLH as proposed by the Histiocyte Society in 2004.^7^ The table below shows the diagnostic criterion for HLH/MAS used in our case.

**Table 1:** Diagnostic criteria for MAS/HLH.^7^

Major criteria^*^ Fever of >38.5° C for atleast 7 days Splenomegaly Cytopenia involving >2 cell lines Hypertriglyceridemia or hypofibrinogenemia Hemophagocytosis demonstrated in bone marrow, spleen, or lymph nodes without evidence of malignancy Alternative criteria^†^ Low or absent natural killer cell activity Serum ferritin level of >500 μg/L Soluble CD25 (soluble IL-2 receptor) level at >2400 U/ml

* A diagnosis of HLH requires the presence of all 5 major criteria. If the patient meets only 4 criteria but the clinical suspicion for HLH is high, one should initiate treatmnet, because delays may be fatal.

† Alternative criteria 1 or a combination of 2 and 3 may substitue for 1 major criterion.

Early recognition and treatment is imperative for management of MAS. The inflammatory cascade is treated promptly with immunosuppression, usually high-dose corticosteroids. Fever subsided after 2 doses of intravenous Methylprednisolone. Cyclosporine has been reported to produce rapid response when used in managemnt of severe cases and also in patients who fail to respond to steroids. Cyclosporine may be chosen as a first line therapy or added to first line steroid treatment. Other treatment options include plasma exchanges, intravenous immunoglobulins and etoposide.^8^

In conclusion, MAS is a serious life threatening condition resulting from hyper-activation of macrophages, which can be associated with SoJIA. The clinical picture of MAS may mimic the flare of SoJIA leading to misdiagnosis . The clinical findings of non-remitting fever, hepatosplenomegaly, lymphadenopathy, blood diathesis, rashes and altered mental status along with laboratory findings of leucopenia and thrombocytopenia should alert the physician of this condition. The elevation of serum ferritin, triglyceride and fibrinogen may assist in prompt diagnosis of MAS. Cyclosporine and Corticosteroids are commonly used drugs for treatment of MAS.
